# Comparison of Diagnostic Models to Estimate the Risk of Metabolic Syndrome in a Chilean Pediatric Population: A Cross-Sectional Study

**DOI:** 10.3390/metabo13020293

**Published:** 2023-02-16

**Authors:** Marlín Solorzano, Gislaine Granfeldt, Natalia Ulloa, Guillermo Molina-Recio, Rafael Molina-Luque, Claudio Aguayo, Fanny Petermann-Rocha, Miquel Martorell

**Affiliations:** 1 Programa de Magíster en Nutrición Humana, Departamento de Nutrición y Dietética, Universidad de Concepción, Concepción 4070386, Chile; 2 Residente del Programa de Endocrinología Adultos, Departamento de Endocrinología, Escuela de Medicina, Universidad Católica de Chile, Santiago 8330077, Chile; 3 Departamento de Nutrición y Dietética, Facultad de Farmacia, Universidad de Concepción, Concepción 4070386, Chile; 4 Departamento de Bioquímica Clínica e Inmunología, Facultad de Farmacia, Universidad de Concepción, Concepción 4070386, Chile; 5 Centro de Vida Saludable, Universidad de Concepción, Concepción 4070386, Chile; 6 Lifestyles, Innovation and Health (GA-16), Maimonides Biomedical Research Institute of Cordoba (IMIBIC), 14004 Córdoba, Spain; 7 Department of Nursing, Pharmacology and Physiotherapy, University of Córdoba, 14004 Córdoba, Spain; 8 Centro de Investigación Biomédica, Facultad de Medicina, Universidad Diego Portales, Santiago 8370068, Chile

**Keywords:** metabolic syndrome, pediatric obesity, anthropometry, metabolic biomarkers, adipokines, early diagnosis

## Abstract

The pediatric population has various criteria for measuring metabolic syndrome (MetS). The diversity of consensus for diagnosis has led to different non-comparable reported prevalence. Given the increase in its prevalence in pediatric ages, it is necessary to develop efficient methods to encourage early detection. Consequently, early screening for the risk of MetS could favor timely action in preventing associated comorbidities in adulthood. This study aimed to establish the diagnostic capacity of models that use non-invasive (anthropometric) and invasive (serum biomarkers) variables for the early detection of MetS in Chilean children. A cross-sectional study was carried out on 220 children aged 6 to 11. Multivariate logistic regressions and discriminant analyses were applied to determine the diagnostic capacity of invasive and non-invasive variables. Based on these results, four diagnostic models were created and compared: (i) anthropometric, (ii) hormonal (insulin, leptin, and adiponectin), (iii) Lipid A (high-density cholesterol lipoprotein [HDL-c] and triglycerides [TG]) and (iv) Lipid B (TG/HDL-c). The prevalence of MetS was 26.8%. Lipid biomarkers (HDL-c and TG) and their ratio (TG/HDL-c) presented higher diagnostic capacity, above 80%, followed by body mass index (BMI, 0.71–0.88) and waist-to-height ratio (WHtR, 0.70–0.87). The lipid model A was the most accurate (sensitivity [S] = 62.7%, specificity [E] = 96.9%, validity index 87.7%), followed by the anthropometric model (S = 69.5%, E = 88.8% and validity index = 83.6%). In conclusion, detecting MetS was possible through invasive and non-invasive methods tested in overweight and obese children. The proposed models based on anthropometric variables, or serum biomarkers of the lipid model A, presented acceptable validity indices. Moreover, they were higher than those that measured adipokines, leptin, and adiponectin. The anthropometric model was the most cost-effective and easy to apply in different environments.

## 1. Introduction

Metabolic syndrome (MetS) is referred to a series of metabolic abnormalities that increase the risk of developing type-2 diabetes mellitus (T2D) and cardiovascular diseases (CVDs). Although it is usually investigated in the adult population, it can also occur in children and adolescents [1,2]. To date, there is no consensus on the definition of MetS for the pediatric population [3,4]. However, most authors agree that in this population, the essential components are glucose intolerance, central obesity, high blood pressure (HBP), and an imbalance in the blood lipid levels (decreased levels of high-density cholesterol lipoprotein [HDL-c] and hypertriglyceridemia) [5,6].

In recent years, the prevalence of MetS has increased exponentially due to multiple causes, among which the presence of obesity is highlighted [7,8]. However, its estimation in children and adolescents has been difficult due to geographic variability and the definitions used [9]. The prevalence of MetS ranges from 3% to 19% in pediatric populations, which increases significantly in the presence of a higher body mass index (BMI), reaching up to 29% [10]. Due to the increased prevalence of MetS in pediatric ages, efforts have been made to develop increasingly efficient methods that encourage its early detection to mitigate the onset of associated comorbidities during adulthood.

Among the different tools for the early detection of MetS, non-invasive or anthropometric ones can be mentioned, as well as those that require invasive techniques such as the measurement of serum biomarkers [11,12]. Different authors have pointed out that anthropometric techniques are the most appropriate because they can be used in a greater variety of clinical settings [11,13,14]. However, during the last decade, great developments have been made in comprehending serum biomarkers such as adipocytokines, including leptin, and adiponectin, which performs a relevant role in energy homeostasis and glucose and lipid metabolism [15]. That is why it has been prioritized to deepen the research of these adipocytokines in the pediatric population. Thus, evidence has been found that these could detect or predict the onset of MetS early [12].

On the other hand, developing diagnostic methods has become increasingly frequent in investigating non-communicable chronic diseases. It is widely recommended for use within clinical practice guidelines to optimize decision-making [16]. The diagnostic models allow knowing whether specific prognostic factors or markers, which are more invasive or expensive, have an added valuable predictive value beyond economic predictors or obtained, for example, from the history of patients or physical examination [17]. In the MetS, it is known that there are physical or anthropometric changes whose measurement is of low cost or, on the other hand, alterations in risk biomarkers, whose measurement may be more expensive in clinical practice.

According to the latest Country Nutrition Profiles reported by the Global Nutrition Report [18], Chile has made much progress in childhood stunting and wasting, having a lower prevalence than the rest of Latina America. However, the prevalence of overweight in children under five years is still higher in Chile than in Latin America (9.3% vs. 7.5%) [18]. Moreover, the Nutritional Map Report 2021 (JUNAEB) highlighted that obesity increased from 17.8% in 2020 to 20.3% in 2021 in Chilean children and adolescents [19]. Moreover, even if there is no national estimation of the MetS in Chilean children, some authors have reported ranges from 4% to 45% [20,21,22,23].

Given the above, and the high prevalence of overweight and obesity in Chilean children, the objective of the present research was to establish diagnostic risk models that use non-invasive and invasive variables for the early detection of MetS in Chilean children.

## 2. Materials and Methods

### 2.1. Design, Population and Sample

A descriptive cross-sectional study was carried out in children aged between 6 and 11 years, who belonged to the urban environment of the city of Hualpén, Biobío Region of Chile. Clinical data and blood sample collection were performed between March and June 2008.

For an estimated prevalence of MetS in children of 22.7% [23], considering an accuracy of 6%, a 95% confidence interval (95% CI), and a total population of 1,556,805 in the Biobío Region (according to the 2017 census [24]), we calculated a minimum sample size of 188 individuals. We decided to use 6% considering this was not a prevalence study but rather an associative model study. Finally, we studied 220 children aged between 6 to 11 years to keep the sample as large as possible considering the project’s budget. Moreover, children suffering from a chronic condition and those whose parents or legal guardians did not sign the informed consent were excluded.

### 2.2. Study Variables and Measurements

The independent variables included were sex (boy and girl), age (years), and those grouped in:

**Anthropometric variables:** weight (kg), height (cm), BMI (kg/m^2^), waist circumference (WC, cm), waist-to-height ratio (WHtR), fat-free mass (FFM, kg), body fat (BF, kg), BF percentage (%), systolic blood pressure (SBP, mmHg), and diastolic blood pressure (DBP, mmHg).

Trained nutritionists performed all anthropometric measurements. Weight and body composition (BF, % of BF and FFM) were measured with a bioimpedance meter (TANITA TBF-300, TANITA, Tokyo, Japan) with an accuracy of 0.1 kg. Height was measured without shoes, using wall-mounted stadiometers to the nearest 0.1 cm (Seca, model 208). WC was measured in the middle point between the lowest rib and the upper edge of the iliac crest, with a noncompliant measuring tape at the nearest 0.1 cm (Seca, model 201). BMI (kg/m^2^) was calculated at the body weight divided by height squared. The BMI z-score based on age and sex was calculated according to the methods recommended by the World Health Organization (WHO) [25]. Children were classified as underweight (BMI 5th percentile), normal (BMI ≥ 5th percentile and <85), overweight (BMI ≥ percentile 85 and <95), or obese (BMI > percentile 95), according to the specific international percentiles by age and gender defined by the Center for Disease Control and Prevention [26]. WHtR was calculated by dividing WC by height, both in centimeters. The figure <0.55 [27] was taken as a normal value. To determine the presence of waist obesity (WC > percentile 90), reference tables developed for the pediatric population by Fernández, et al. [28] were used according to age and sex were used.

A physician measured blood pressure through a calibrated digital sphygmomanometer (OMRON M3, OMRON, Kyoto, Japan). The cuff length to measure blood pressure was chosen according to the arm circumference. Children were asked to sit down for at least five minutes before measurement. Two records were taken with an interval of two minutes between them, where average SBP and DBP were recorded. To determine the presence of HBP (SBP o DBP > percentile 90), reference tables were used, according to age, gender, and height [29].

**Metabolic and hormonal variables:** Glucose (mg/dL), HDL-c (mg/dL), LDL-c (mg/dL), triglycerides (TG, mg/dL), total cholesterol (TC, mg/dL), insulin (μU/mL), adiponectin (μg/mL) and leptin (ng/mL) were considered.

For metabolic and hormonal analyses, 4 mL of venous blood was drawn after an overnight fast of 8 to 12 h. Serum concentrations of TC, HDL-c, LDL-c, TG, and glycemia levels were determined through colorimetric methods, using commercially available kits (Cobas C111 Roche, Indianápolis, IN, USA). Plasmatic insulin and adiponectin were measured using an ELISA commercial kit (Linco Research, St. Charles, MO, USA) in a Synergy 2 multi-mode reader (Biotek, Winooski, VT, USA). Based on the ELISA technique, leptin was determined through a commercial immunoassay kit (Quantikine R&D Systems, Minneapolis MN, USA) in plates sensitized with the human anti-leptin monoclonal antibody. The HOMA-IR index was calculated from the previously defined baseline glucose and insulin concentrations [30]. Experienced technicians carried out all measurements to minimize the variation coefficient. A test was considered abnormal when the glucose levels were ≥100 mg/dL, TC ≥ 200 mg/dL, HDL-c ≤ 40 mg/dL, LDL-c ≥ 100 mg/dL, or TG ≥ 110 mg/dL. Regarding the reference values of insulin, leptin, and adiponectin, predetermined cut-off points were not considered because they are unknown for the pediatric population studied. Instead, they were established according to the discriminant capacity for MetS detected in this study.

### 2.3. Diagnosis of Metabolic Syndrome

The MetS diagnosis was determined by at least 3 out of 5 components of the Cook phenotype [31]. The cut-off points used were WC ≥ 90th percentile; percentile of increased blood pressure, either SBP or DBP ≥ 90th percentile; HDL-c ≤ 40 mg/dL; TG ≥ 110 mg/dL; and glycemia ≥ 100 mg/dL.

### 2.4. Ethical Aspects

The study was carried out in compliance with the fundamental principles of the Helsinki Declaration (1964), the Council of Europe Convention on Human Rights and Biomedicine (1997), in the Universal Declaration of UNESCO on the human genome and human rights (1997), as well as complying with the requirements established in Chilean legislation in the field of biomedical research, the protection of personal data and bioethics, according to Decree No. 114, of 2010 that approves the Regulation of Law No. 20,120, and which was modified and updated in Decree 30 of 14 January 2013. Additionally, this study was approved by the Ethics Committee of the Vice-Rectory of Research of the University of Concepción (352-2019). Parents were given written informed consent before their children were included in the study.

### 2.5. Statistical Analyses

Quantitative variables are presented with mean and standard deviation, whereas qualitative variables are presented in frequencies and percentages. To test the goodness of fit to a normal distribution of data from quantitative variables, the Kolmogorov–Smirnov test with Lilliefors correction was used. For the contrast of bivariate hypotheses, the Student’s t-test was used for two means and for non-parametric versions, the Mann–Whitney U-test was used (considering Levene’s test for homogeneity contrast). The area under the curve (AUC) was calculated to determine the diagnostic accuracy of each variable and to establish which best predicted MetS. Sensitivity, specificity, Youden, and validity indices were also analyzed to determine their best cut-off value for greater diagnostic accuracy.

To assess the aptitude of the studied variables (BMI, SBP, DBP, WHtR, HDL-c, TG, TG/HDL-c, insulinemia, leptinemia, adiponectinemia) to predict MetS, binary adjusted methods adjusted by age and gender were performed. The adjusted odds ratio (OR) was determined with its 95% CI. The goodness of fit tests (−2 log likelihood, goodness of fit statistic, Cox and Snell R2, Nagelkerke R2 and Hosmer–Lemeshow tests) were calculated to evaluate the global adjustment of each model.

For all statistical analyses, an alpha error probability of less than 5% (*p* < 0.05) was accepted, and the 95% CIs were calculated. For statistical analyses, the software IBM SPSS Statistics version 22.0 (IBM, Chicago, IL, USA) and EPIDAT version 4.2. (Departamento de Sanidade, Xunta de Galicia, Galicia, Spain) were used.

## 3. Results

### 3.1. Sample Description

Both clinical and anthropometric characteristics for children with and without MetS are described in Table 1. The mean age was 9.1 (1.3) years, without significant differences between boys and girls. Regarding the prevalence of MetS, this was 26.8% (34.5% in girls and 19.1% in boys; *p* < 0.05), with a significantly higher proportion in girls than in boys (OR = 2.2; *p* < 0.05). Except for age, glycemia, and LDL-c, the other variables showed a significant bivariate association with MetS, according to the modified Cook criteria (*p* < 0.001). Children with MetS showed higher levels of risk biomarkers, such as TG, insulin and leptin, and lower levels of HDL-c and adiponectin compared with children without MetS (*p* < 0.001).

### 3.2. Components of the Metabolic Syndrome

Table 2 shows the trends of the mean values for each of the variables included in the study, distributed according to the presence of MetS components (0, 1, 2, and ≥3). Subjects with more MetS components had higher BMI, WC, fat mass, TG, LDL-c, insulin, HOMA-IR, SBP, DBP, and leptin, and lower levels of HDL-c and adiponectin (*p* < 0.001). When evaluating the linearity of the ratio between the variables under study and the number of MetS components, the mean of the values of both serum biomarkers (TG, insulin, and leptin) and the anthropometric variables (WC, BMI and fat mass) increased progressively and continuously as more MetS components were added. On the other hand, HDL-c and adiponectin decreased progressively in the function of the number of MetS components presented. The SBP increased by 5 mmHg for each component of the added MetS.

### 3.3. MetS Diagnostic Accuracy and Discriminant Capacity of the Study Variables

When evaluating the discriminant capacity of each diagnostic variable, it was found that lipid biomarkers (HDL-c and TG), and their ratio (TG/HDL-c), have a greater diagnostic capacity, measured by AUC (above 80%), followed by WC (0.80 [0.72–0.88)], BMI (0.79 [0.71–0.88]) and WHtR (0.78 [0.70–0.87]), as shown in Table 3.

### 3.4. Risk Diagnostic Models

Considering the variables analyzed above that presented a statistically significant association, and avoiding including those that could generate *a priori* high collinearity, four diagnostic models were proposed and compared for MetS in children (Table 4). For all models, variables were dichotomized (except for gender in the anthropometric model), depending on whether the value is higher or lower than the cut-off value shown in Table 3.

The results of the indicators (sensitivity, specificity, and validity index) are presented in Table 4. This allowed knowing the diagnostic performance of the four models after being adjusted by age and sex. Although all the models presented can be valid to estimate MetS, the Lipid model A, based only on the determination of HDL-c (OR= 19.3, 95% CI: 8–46.7; *p* <0.001) and TG (OR= 8.0, 95% CI: 3.1–20.1; *p* < 0.001) turned out to be the most accurate.

## 4. Discussion

A prevalence of 26.8% in the MetS was found in the pediatric sample, which was significantly higher than that found by other authors, possibly due to the diversity of criteria used to define MetS and the characteristics of the populations studied [32,33]. However, this was similar to that described in the IDEFIC study [6] and in two other studies carried out in Chilean pediatric populations, which also used the Cook criteria to define MetS [23,34]. On the other hand, it is recognized that overweight and obesity are the most important features contributing to the increased presence of MetS in children [21,35,36]. This is consistent with the results of the present study, since 96.6% of the children included with MetS presented overweight or obesity.

When describing the characteristics of the cases that presented MetS, it was evidenced that all variables—except age, glycemia, TC, and LDL-c—showed a significant bivariant association with MetS. The lack of association with fasting glycemia reflects the existence of the known initial hyper-insulinemia compensatory mechanism [37]. The baseline insulin levels and the HOMA-IR index were higher in children who presented MetS, which is consistent with the findings of another Chilean study [29] and other populations [38,39]. There was a linear association between the variables under study and the number of MetS components. Moreover, the mean of the values of the variables increased, both in the non-invasive (BMI, WHtR, WC, SBP) and in the invasive (LDL-c, TG, glucose, leptin), or decreased (adiponectin) progressively and continuously in the function of the number of MetS components presented by the children. This allows the possibility to recognize the development toward MetS and, therefore, act early.

The greatest diagnostic capacity of MetS found in the present study was given by non-invasive variables (anthropometric and blood pressure). Among the anthropometric variables, the BMI stands out (AUC = 0.79, 95% CI: 0.71–0.88), with a cut-off point of 23.5 kg/m^2^. Despite not considering body composition or fat distribution, this index suggests other adiposity indices in predicting MetS; therefore, it should be the choice considering its ease of calculation [40]. Other authors also demonstrated that BMI has good diagnostic power for MetS [41,42]. Along the same line, several studies highlight the WC for predicting MetS [43,44,45], which can add more information to that provided by the BMI [45]. Regarding WHtR, a value ≥0.55 significantly increased the risk of suffering MetS in this study. In addition, it had a moderate diagnostic capacity, with an AUC = 0.78 (95% CI: 0.70–0.87), being similar to the results of a meta-analysis in which the WHtR, as a quantitative measurement, showed an AUC = 0.76 (95% CI: 0.71–0.80). Thus, WHtR could be considered in the periodic health check-ups of children and adolescents since it can measure the risk of MetS, independently of the degree of general obesity [46].

In obesity and MetS, the presence of dysfunctional adipocytes is described. These cells present, among others, alterations in the secretion of adipokines, such as a reduction of adiponectin and increased leptins [47,48,49,50]. Indeed, the adiponectin levels of children in this study who presented MetS were significantly lower, and the leptin levels were higher than those who lacked MetS (*p* < 0.001). The reduction in adiponectin and increase in leptin was inversely proportional as more MetS components were added, similar to what was found in previous reports [42,43]. This supports the idea that adipokines could be a good biomarker to identify individuals at risk of presenting MetS [51,52]. Likewise, this study found that both adipokines present a moderate diagnostic capacity, similar to the results described in a meta-analysis carried out in an Asian population [53]. At present, it is unclear which serum adipokine value is more appropriate to identify MetS in the pediatric population. The cut-off value of leptin found in this study to discriminate the presence of MetS was 14.00 ng/mL, similar to that found by Madeira, et al. [54] in prepubertal children, with an optimal leptin cut-off point of 13.4 ng/mL. This data can be beneficial to interpret this biomarker’s result in Chilean children adequately.

Dyslipidemia is related to MetS and its onset at an early age can predict future cardiovascular complications. The results of the present study show an atherogenic profile in the MetS group, with higher LDL-c, lower HDL-c, higher TG, and higher values of the TG/HDL-c ratio, with statistically significant differences between groups with and without MetS. This result is consistent with previous reports [55,56]. Therefore, children with MetS have an atherogenic lipid profile that should be examined and intervened to correct early. Indeed, in a pediatric population with MetS, it has been reported a reversion of lipid alteration [57]. In addition, the variables of this study with greater diagnostic capacity are two lipid variables: TG and HDL-c, as well as their ratio (TG/HDL-c), all with an AUC > 80%. This is consistent with the findings by Liang, et al. [58], in which the TG/HDL-c ratio was the best indicator for MetS, with a larger AUC (AUC = 0.84). However, a much lower cut-off value compared to that of the present study >1.25 vs. >2.33, respectively, was found. The latter is expected since there is a cut-off value defined by the TG/HDL-c ratio in the children population, which may vary depending on the population analyzed. A more recent study also found that the TG/HDL-c ratio is an early predictor of MetS and strongly correlated with their components. Thus, it can be used as an effective index in children, regardless of age and predisposition to MetS [59]. In a study performed in men and women (*n* = 797, 35–60 years) of Aboriginal, Chinese, European, and South Asian origin, the TG/HDL-c ratio was a superior indicator for MetS compared to TC/HDL-c, LDL-c/HDL-c, and nonHDL-c/HDL-c [60]. Moreover, TG/HDL-c ratio has been proposed as an indirect measure of small dense LDL, which is the most atherogenic LDL fraction [61,62]. Thus, TG/HDL-c ratio seems a reliable indicator for MetS and CVDs.

Four diagnostic models were proposed and compared based on the findings described, centered on invasive and non-invasive variables, which were statistically significant in the logistic regression analysis. These allowed the early detection of MetS. All the models presented can be valid to predict MetS, though lipid model A, based on the determination of HDL-c and TG, turned out to be the most accurate to predict MetS (sensitivity of 62.7%, specificity of 96.9%, and validity index of 87.7%). Although an invasive technique is involved (blood sample extraction), its determination is widely used in clinical practice. This technique is also cheaper and, therefore, more efficient than the hormonal model, which requires the analysis of insulin, leptin, and adiponectin, which are not always available in the clinical laboratory routine and are of high cost. The lipid model B (TG/HDL-c) also showed high sensitivity, reaching 83.3%, which could facilitate the screening of children with LBP with MetS. With this point of view, oxidative stress markers may be used to develop new diagnostic models to estimate the risk of MetS [63] but with associated high costs.

For a long time, anthropometric measurements have been recognized and widely used as convenient indicators in the prediction of MetS. In addition, the data found in this study show a fundamental role of BMI, WC, WHtR, and blood pressure in the early non-invasive diagnosis of MetS. Although in other populations, WC and WHtR have been proposed as an alternative to BMI in the discrimination of MetS [64,65], results found in the present research have shown that its combined use (WHtR and BMI) in the non-invasive model together with the measurement of SBP and DBP can increase its diagnostic capacity (S = 69.5%, E = 88.8% and validity index = 83.6%). The second one is the most accurate of the four models proposed. This model has a notable advantage since it could be applied in any context (e.g., school, primary care, etc.,) and has good diagnostic precision and the possibility of reducing the blood draws involved, especially in places with scarce health resources, capable of being more cost-effective.

Early detection of MetS is difficult in clinical settings due to the absence of a universal definition and variability of components and their cut-off points. Hence, the data provided by this study can be very useful in understanding the behavior of the different MetS components in Chilean children and also provide additional information regarding emerging and promising metabolic risk biomarkers such as adiponectin and leptin. Of the four diagnostic models proposed, the one that includes non-invasive techniques (anthropometric model) makes it an inexpensive option that could be easy to apply in any healthcare setting or even schools. This model may facilitate the early detection of children at risk in the school environment, their timely referral to primary care, and the application of early interventions, preventing the possible complications associated with MetS in the following stages of life.

### Strengths and Limitations

Among the strengths of this study, we can highlight that the nutritional evaluation and blood pressure of the children were carried out by highly trained personnel and that, according to the available evidence, this work and the models proposed seem to be the first tools to predict MetS in Chilean children. However, this study is not exempt from limitations. First, due to the cross-sectional nature of the study, cause-and-effect associations cannot be established. Second, the sample of children analyzed had a high prevalence of overweight and obesity, which raises the prevalence of MetS, above that shown in the pediatric population. Third, given the high prevalence of obesity in the sample, the models had to match the population used in the study closely. For this reason, future work should consider samples with a better representation and distribution of the nutritional status. Fourth, considering the heterogeneity of diagnostic criteria and the scarce consensus around them, the modified Cook criteria were used. These are widely distributed to facilitate the comparison with other works carried out in similar populations. Fifth, given the pubertal changes that can influence anthropometric results [66] and risk biomarkers, due to the physiological insulin resistance described in this stage of life [67], further studies are suggested to consider this variable. Sixth, due to the number of variables in the study, it was impossible to collect all possible models that could be run. The latter was associated with methodological unfeasibility (non-compliance with the assumptions of the logistic regression model) and coefficient of determination (validity indices and/or other statistics with low values of no scientific or clinical interest). Therefore, only the four models already mentioned met all the methodological and clinical relevance criteria and were included in the study. Finally, this study did not make a comparison with other scores, such as those used for the NAFLD diagnoses or the fibrosis-4 index (Fib 4) [68], since some biochemical measures were not available for the analytes in the samples (e.g., platelet count).

## 5. Conclusions

The prevalence of MetS found in this pediatric population was high, especially among children who were overweight and obese. The model based on non-invasive variables presented good diagnostic accuracy and could be easy to apply in different settings (school or health). However, according to the results found, HDL-c and TG are the variables with greater diagnostic capacity. The TG/HDL-c included in the proposed lipid models turned out to be the most accurate for the early detection of MetS. These parameters can be obtained in the primary care setting, and when compared with other biomarkers such as leptin and adiponectin, they are inexpensive. For this reason, it is suggested not to rule out its use if they are available. Both models can be convenient and beneficial because they allow not only to perform an early diagnosis, but a timely derivation which enables the implementation of interventions that help correct the different disorders found in MetS and prevent the onset of cardiovascular or metabolic complications in adulthood.

## Figures and Tables

**Table 1:** Characteristics of the sample according to the presence of MetS.

Variable	Total *n* = 220 Mean (SD or %)	With MetS *n*= 59 Mean (SD or %)	Without MetS *n* = 161 Mean (SD or %)	*p*
BOYS	110 (50%)	21 (19.1%)	89 (80.9%)	<0.05
GIRLS	110 (50%)	38 (34.5%)	72 (65.5%)	
AGE (years)	9.1 (1.3)	9.3 (1.2)	9 (1.3)	0.13
WC (cm)	74.5 (11.6)	83.6 (8.1)	71.2 (10.9)	<0.001
WHtR (≥0.55)	128 (57.9)	55 (93.2)	73 (45.1)	<0.001
BMI (kg/m^2^)	22.2 (4.2)	25.6 (3.1)	21 (3.8)	<0.001
Overweight + obesity	148 (67)	57 (96.6)	91 (56.2)	<0.001
BF (kg)	13.3 (6.7)	18.3 (5.7)	11.5 (6.2)	<0.001
%BF	30.1 (9.5)	36.6 (6)	27.6 (9.4)	<0.001
FFM (kg)	28.4 (5.1)	31.1 (5.1)	27.4 (4.7)	<0.001
SBP (mmHg)	101.6 (12)	108.7 (12)	98.9 (10.9)	<0.001
DBP (mmHg)	66.1 (10.6)	71.1 (10.7)	64.2 (10)	<0.001
GLYCEMIA (mg/dL)	88.3 (8.6)	88.6 (10)	88.2 (7.9)	0.77
TC (mg/dL)	181.8 (33.1)	188.2 (36.4)	179.5 (31.6)	0.09
HDL-c (mg/dL)	50.5 (11.7)	40.4 (10.2)	54.2 (89.8)	<0.001
LDL-c (mg/dL)	108.3 (28.9)	113.3 (32.9)	106.4 (27.1)	0.12
TG (mg/dL)	102.3 (84.2)	173.9 (119.8)	88 (63.1)	<0.001
TG/HDL-c	2.8 (2.5)	5.2 (3.6)	1.9 (1.1)	<0.001
Baseline insulin (μU/mL)	8.6 86.8)	12.4 (6.9)	7.2 (6.3)	<0.001
HOMA-IR	1.9 (1.4)	2.7 (1.4)	1.6 (1.2)	<0.001
Adiponectinemia (μg/mL)	15 (5.6)	12.3 (3.7)	16 (5.9)	<0.001
Leptinemia (ng/mL)	17.2 (10.7)	23.9 (9.1)	14.0 (9.7)	<0.001

MetS, metabolic syndrome; SD, standard deviation; WC, waist circumference; WHtR, waist-to-height ratio index; BMI, body mass index; BF, body fat; FFM, fat-free mass; SBP, systolic blood pressure; DBP, diastolic blood pressure; TC total cholesterol; HDL-c high-density lipoprotein cholesterol; LDL-c, low density cholesterol lipoprotein cholesterol; TG, triglycerides: TG/HDL, triglycerides/high-density cholesterol lipoprotein ratio; HOMA-IR, homeostasis model for insulin resistance: Statistical analyses: Student’s T-test (for parametric data), Mann–Whitney U test (non-parametric data).

**Table 2:** MetS components and linear trend analysis for independent variables.

Variable	0 Components *n*= 51 Mean (SD or %)	1 Component *n*= 53 Mean (SD or %)	2 Components *n*= 57 Mean (SD or %)	≥3 Components *n*= 59 Mean (SD or %)	r	*p*
WC (cm)	62.3 (6.2)	71.3 (9.8)	79.2 (8.7)	83.6 (8.1)	0.70	<0.001
WHtR (≥ 0.55)	0.48 (0.03)	0.53 (0.07)	0.58 (0.05)	0.6 (0.05)	0.68	<0.001
BMI (kg/m^2^)	17.8 (2.0)	20.9 (3.0)	23.9 (3.4)	25.6 (3.1)	0.71	<0.001
BF (kg)	6.5 (2.9)	11.3 (5.3)	15.8 (5.7)	18.3 (5.7)	0.66	<0.001
%BF	20.1 (5.6)	27.9 (9.4)	33.7 (7.1)	36.6 (6.0)	0.66	<0.001
FFM (kg)	24.5 (84.5)	27.4 (3.8)	29.7 (84.3)	31.1 (5.1)	0.49	<0.001
SBP (mmHg)	93.8 (9.8)	98.6 (10.0)	103.8 (10.5)	108.7 (12.0)	0.47	<0.001
DBP (mmHg)	60.1 (8.3)	65.4 (9.6)	66.9 (10.8)	71.1 (10.7)	0.37	<0.001
HDL-c (mg/dL)	58.8 (9.6)	53.6 (8.0)	50.7 (10.1)	40.4 (10.2)	0.58	<0.001
TG (mg/dL)	66.3 (29.6)	82.8 (48.4)	132.7 (67.0)	173.9 (119.8)	0.61	<0.001
TG/HDL	1.2 (0.4)	1.7 (0.81)	2.8 (1.2)	5.1 (3.6)	0.61	<0.001
Baseline insulinemia (μU/mL)	5.1 (2.8)	6.7 (4.6)	9.5 (8.8)	12.5 (6.9)	0.41	<0.001
HOMA-IR	1.1 (0.65)	1.5 (1.1)	2.0 (1.5)	2.7 (1.4)	0.44	<0.001
Adiponectinemia (μg/mL)	17.3 (5.6)	17.0 (6.4)	13.8 (4.9)	12.3 (3.7)	0.38	<0.001
Leptinaemia (ng/mL)	8.0 (4.2)	12.6 (8.8)	20.2 (10.6)	23.9 (9.1)	0.59	<0.001

MetS, metabolic syndrome; SD, standard deviation; WC, waist circumference; WHtR, waist height index; BMI, body mass index; BF, body fat; FFM, fat-free mass; SBP, systolic blood pressure; DBP, diastolic blood pressure; LDL-c, low density cholesterol lipoprotein; TG, triglycerides: TG/HDL, triglycerides/high-density cholesterol lipoprotein ratio; HOMA-IR, homeostasis model for insulin resistance: Statistical analyses: correlation coefficient of the linear model, value of r.

**Table 3:** Discriminant capacity and MetS diagnostic accuracy for the study variables.

Variable	AUC 95% CI	*p*	Cut-off Values	Youden Index
WC (cm)	0.80 (0.72–0.88)	<0.001	77.50	0.51
WHtR (≥0.55)	0.78 (0.70–0.87)	<0.001	0.53	0.53
BMI (kg/m^2^)	0.79 (0.71–0.88)	<0.001	23.50	0.55
%BF	0.78 (0.69–0.87)	<0.001	30.40	0.50
BF (kg)	0.79 (0.71–0.88)	<0.001	10.25	0.46
FFM (kg)	0.71 (0.61–0.82)	<0.001	29.75	0.37
SBP (mmHg)	0.73 (0.62–0.74)	<0.001	109.50	0.40
DBP (mmHg)	0.70 (0.59–0.81)	<0.001	64.50	0.33
HDL-c (mg/dL)	0.85 (0.77–0.95)	<0.001	41.30	0.68
TG (mg/dL)	0.81 (0.73–0.90)	<0.001	110.20	0.53
TG/HDL	0.87 (0.80–0.95)	<0.001	2.33	0.59
Baseline insulinemia (μU/mL)	0.77 (0.68–0.87)	<0.001	7.63	0.46
HOMA-IR	0.77 (0.68–0.88)	<0.001	1.56	0.42
Adiponectinemia (μg/mL)	0.71 (0.61–0.81)	<0.001	16.95	0.39
Leptinaemia (ng/mL)	0.78 (0.69–0.87)	<0.001	14.00	0.49

MetS, metabolic syndrome; SD, standard deviation; WC, waist circumference; WHtR, waist height index; BMI, body mass index; BF, body fat; FFM, fat-free mass; SBP, systolic blood pressure; DBP, diastolic blood pressure; LDL-c, low density cholesterol lipoprotein; TG, triglycerides: TG/HDL, triglycerides/high-density cholesterol lipoprotein ratio; HOMA-IR, homeostasis model for insulin resistance; AUC, the area under the curve. Statistical analyses: the area under the curve and Youden index were calculated for dichotomic qualitative variables. 95% CI: 95% confidence intervals.

**Table 4:** Logistic regression models adjusted by age and gender.

Anthropometric Model (Non-Invasive)			
Variable	OR (Adjusted) 95% CI	*p*	The Goodness of Fit and Diagnostic Accuracy of the Model
Boys	1 (reference)	<0.01	Hosmer–Lemeshow (*p* > 0.05) R^2^ (Nagelkerke) = 0.48 S = 69.5% E = 88.8% Validity index = 83.6% YI = 0.583
Girls	3.7 (1.6–8.4)		
BMI < 23.50 (kg/m^2^)	1 (reference)	<0.01	
BMI ≥ 23.50 (kg/m^2)^	5.9 (2.1–16.6)		
WHtR < 0.53	1 (reference)	<0.05	
WHtR ≥ 0.53	5.6 (1.3–23)		
SBP < 109.50 (mmHg)	1 (reference)	<0.05	
SBP ≥ 109.50 (mmHg)	2.2 (1.01–4.9)		
DBP < 64.50 (mm/Hg)	1 (reference)	<0.05	
DBP ≥ 64.50 (mm/Hg)	3.1 (1.2–7.7)		
**Lipid Model A**			
HDL-c ≥ 41.30 (mg/dL)	1 (reference)	<0.001	Hosmer–Lemeshow (*p* > 0.05) R^2^ (Nagelkerke) = 0.55 S = 62.7% E = 96.9% Validity index = 87.7% YI = 0.596
HDL-c < 41.30 (mg/dL)	19.3 (8–46.7)		
TG < 110.20 (mg/dL)	1 (reference)	<0.001	
TG ≥ 110.20 (mg/dL)	8.0 (3.1–20.1)		
**Lipid Model B**			
TG/HDL-c < 2.33	1 (reference)	<0.001	Hosmer–Lemeshow (*p* < 0.05) R^2^ (Nagelkerke) = 0.35 S = 83.3% E = 74.5% Validity index = 76.7% YI = 0.578
TG/HDL-c ≥ 2.33	14.6 (6.6–32.5)		
**Hormonal Model**			
Insulinemia < 7.63 (μU/mL)	1 (reference)	<0.01	Hosmer–Lemeshow (*p* > 0.05) R^2^ (Nagelkerke) = 0.49 S = 68.6% E = 85.1% Validity index = 79.8% YI = 0.537
Insulinemia ≥ 7.63 (μU/mL)	5.0 (1.7–15.1)		
Leptinaemia < 14.00 (ng/mL)	1 (reference)	<0.05	
Leptinaemia ≥ 14.00 (ng/mL)	6.0 (1.5–23.9)		
Adiponectinemia ≥ 16.95 (μg/mL)	1 (reference)	<0.05	
Adiponectinemia < 16.95 (μg/mL)	9.1 (1.8–46.6)		

OR, odds Ratio; BMI, body mass index; WHtR, waist height ratio; SBP, systolic blood pressure; DBP, diastolic blood pressure; HDL-c, high-density cholesterol lipoprotein; TG, triglycerides; TG/HDL, triglyceride/lipoprotein ratio of high-density cholesterol; S, sensitivity; E, specificity; YI, Youden index. Statistical analyses: logistic regression models adjusted for age and sex, Nagelkerke R2 and Hosmer–Lemeshow tests for global model fit.

## Data Availability

The data presented in this study are available on request from the corresponding author. Data is not publicly available due to privacy or ethical restrictions.

## References

[1] Bussler S., Penke M., Flemming G., Elhassan Y.S., Kratzsch J., Sergeyev E., Lipek T., Vogel M., Spielau U., Körner A. (2017). Novel Insights in the Metabolic Syndrome in Childhood and Adolescence. Horm. Res. Paediatr..

[2] Samson S.L., Garber A.J. (2014). Metabolic syndrome. Endocrinol. Metab. Clin. North Am..

[3] Magge S.N., Goodman E., Armstrong S.C., Nutrition C.O., Endocrinology S.O., Obesity S.O. (2017). The Metabolic Syndrome in Children and Adolescents: Shifting the Focus to Cardiometabolic Risk Factor Clustering. Pediatrics.

[4] Titmuss A.T., Srinivasan S. (2016). Metabolic syndrome in children and adolescents: Old concepts in a young population. J. Paediatr. Child. Health.

[5] Al-Hamad D., Raman V. (2017). Metabolic syndrome in children and adolescents. Transl. Pediatr..

[6] Ahrens W., Moreno L.A., Mårild S., Molnár D., Siani A., De Henauw S., Böhmann J., Günther K., Hadjigeorgiou C., Iacoviello L. (2014). Metabolic syndrome in young children: Definitions and results of the IDEFICS study. Int. J. Obes..

[7] Engin A. (2017). The Definition and Prevalence of Obesity and Metabolic Syndrome. Adv. Exp. Med. Biol..

[8] Kim E.G., Kaelber D.C. (2022). Phenotypic prevalence of obesity and metabolic syndrome among an underdiagnosed and underscreened population of over 50 million children and adults. Front. Genet..

[9] Christian Flemming G.M., Bussler S., Körner A., Kiess W. (2020). Definition and early diagnosis of metabolic syndrome in children. J. Pediatr. Endocrinol. Metab..

[10] Friend A., Craig L., Turner S. (2013). The prevalence of metabolic syndrome in children: A systematic review of the literature. Metab. Syndr. Relat. Disord..

[11] Mastroeni S.S.B.S., Mastroeni M.F., Ekwaru J.P., Setayeshgar S., Veugelers P.J., Gonçalves M.C., Rondó P.H.C. (2019). Anthropometric measurements as a potential non-invasive alternative for the diagnosis of metabolic syndrome in adolescents. Arch. Endocrinol. Metab..

[12] Nappo A., González-Gil E.M., Ahrens W., Bammann K., Michels N., Moreno L.A., Kourides Y., Iacoviello L., Mårild S., Fraterman A. (2017). Analysis of the association of leptin and adiponectin concentrations with metabolic syndrome in children: Results from the IDEFICS study. Nutr. Metab. Cardiovasc. Dis..

[13] Romero-Saldaña M., Fuentes-Jiménez F.J., Vaquero-Abellán M., Álvarez-Fernández C., Molina-Recio G., López-Miranda J. (2016). New non-invasive method for early detection of metabolic syndrome in the working population. Eur. J. Cardiovasc. Nurs..

[14] Arsang-Jang S., Kelishadi R., Esmail Motlagh M., Heshmat R., Mansourian M. (2019). Temporal Trend of Non-Invasive Method Capacity for Early Detection of Metabolic Syndrome in Children and Adolescents: A Bayesian Multilevel Analysis of Pseudo-Panel Data. Ann. Nutr. Metab..

[15] Friedman J.M. (2011). Leptin and the regulation of body weigh. Keio. J. Med..

[16] Riley R.D., Windt D.v.d., Croft P., Moons K.G.M. (2019). Prognosis Research in Healthcare: Concepts, Methods, and Impact.

[17] Moons K.G., Royston P., Vergouwe Y., Grobbee D.E., Altman D.G. (2009). Prognosis and prognostic research: What, why, and how?. BMJ.

[18] Report G.N. Country Nutrition Profile. https://globalnutritionreport.org/resources/nutrition-profiles/latin-america-and-caribbean/south-america/chile/.

[19] JUNAEB Informe Mapa Nutricional 2021. https://www.junaeb.cl/wp-content/uploads/2022/10/INFORME-MAPA-NUTRICIONAL-2021_FINAL.pdf.

[20] Burrows A.R., Leiva B.L., Weistaub G., Ceballos S.X., Gattas Z.V., Lera M.L., Albala B.C. (2007). Prevalence of metabolic syndrome in a sample of Chilean children consulting in an obesity clinic. Rev. Med. Chil..

[21] Mardones F., Arnaiz P., Barja S., Giadach C., Villarroel L., Domínguez A., Castillo O., Farias M. (2013). Nutritional status, metabolic syndrome and insulin resistance in children from Santiago (Chile). Nutr. Hosp..

[22] Burrows R., Correa-Burrows P., Reyes M., Blanco E., Albala C., Gahagan S. (2016). High cardiometabolic risk in healthy Chilean adolescents: Associations with anthropometric, biological and lifestyle factors. Public. Health Nutr..

[23] Eyzaguirre F., Silva R., Román R., Palacio A., Cosentino M., Vega V., García H. (2011). Prevalence of metabolic syndrome in children and adolescents who consult with obesity. Rev. Med. Chil..

[24] Censo de Población y Vivienda 2017. http://resultados.censo2017.cl/.

[25] de Onis M., Habicht J.P. (1996). Anthropometric reference data for international use: Recommendations from a World Health Organization Expert Committee. Am. J. Clin. Nutr..

[26] Kuczmarski R.J., Ogden C.L., Grummer-Strawn L.M., Flegal K.M., Guo S.S., Wei R., Mei Z., Curtin L.R., Roche A.F., Johnson C.L. (2000). CDC growth charts: United States. Adv. Data.

[27] Arnaiz P., Acevedo M., Díaz C. (2010). Razón cintura estatura como predictor de riesgo cardiometabólico en niños y adolescentes. Rev. Chil. Cardiol..

[28] Fernández J.R., Redden D.T., Pietrobelli A., Allison D.B. (2004). Waist circumference percentiles in nationally representative samples of African-American, European-American, and Mexican-American children and adolescents. J. Pediatr..

[29] MINSAL (2014). Orientaciones técnicas para el control de salud integral de adolescentes. Control Joven Sano.

[30] Matthews D.R., Hosker J.P., Rudenski A.S., Naylor B.A., Treacher D.F., Turner R.C. (1985). Homeostasis model assessment: Insulin resistance and beta-cell function from fasting plasma glucose and insulin concentrations in man. Diabetologia.

[31] Cook S., Weitzman M., Auinger P., Nguyen M., Dietz W.H. (2003). Prevalence of a metabolic syndrome phenotype in adolescents: Findings from the third National Health and Nutrition Examination Survey, 1988-1994. Arch. Pediatr. Adolesc. Med..

[32] Guzmán-Guzmán I.P., Salgado-Bernabé A.B., Muñoz Valle J.F., Vences-Velázquez A., Parra-Rojas I. (2015). Prevalence of metabolic syndrome in children with and without obesity. Med. Clin..

[33] Ye P., Yan Y., Ding W., Dong H., Liu Q., Huang G., Mi J. (2015). Prevalence of metabolic syndrome in Chinese children and adolescents: A Meta-analysis. Zhonghua Liu Xing Bing Xue Za Zhi.

[34] Bustos P., Orias J., Sáez K., Maldonado M., Cuadra L., Asenjo S. (2015). Effects of the Bright Bodies Program in Chilean obese children. Rev. Med. Chil..

[35] Gepstein V., Weiss R. (2019). Obesity as the Main Risk Factor for Metabolic Syndrome in Children. Front. Endocrinol..

[36] Sapunar J., Aguilar-Farías N., Navarro J., Araneda G., Chandia-Poblete D., Manríquez V., Brito R., Cerda A. (2018). High prevalence of overweight, obesity, insulin resistance and metabolic syndrome in rural children and adolescents. Rev. Med. Chil..

[37] Ten S., Maclaren N. (2004). Insulin resistance syndrome in children. J. Clin. Endocrinol. Metab..

[38] Arellano-Ruiz P., García-Hermoso A., Cavero-Redondo I., Pozuelo-Carrascosa D., Martínez-Vizcaíno V., Solera-Martinez M. (2019). Homeostasis Model Assessment cut-off points related to metabolic syndrome in children and adolescents: A systematic review and meta-analysis. Eur. J. Pediatr..

[39] Cho J., Hong H., Park S., Kim S., Kang H. (2017). Insulin Resistance and Its Association with Metabolic Syndrome in Korean Children. Biomed. Res. Int..

[40] Radetti G., Fanolla A., Grugni G., Lupi F., Sartorio A. (2019). Indexes of adiposity and body composition in the prediction of metabolic syndrome in obese children and adolescents: Which is the best?. Nutr. Metab. Cardiovasc. Dis..

[41] Jung C., Fischer N., Fritzenwanger M., Figulla H.R. (2010). Anthropometric indices as predictors of the metabolic syndrome and its components in adolescents. Pediatr. Int..

[42] Lee K. (2019). Comparison of Body Mass Index Percentiles to Detect Metabolic Syndrome Using the Korean, United States Centers for Disease Control and Prevention, and World Health Organization References in Korean Children Aged 10-16 Years. Metab. Syndr. Relat. Disord..

[43] Oliveira R.G., Guedes D.P. (2018). Performance of anthropometric indicators as predictors of metabolic syndrome in Brazilian adolescents. BMC Pediatr..

[44] Andaki A.C.R., Mendes E.L., Santos A., Brito C.J., Tinôco A.L.A., Mota J. (2018). Waist circumference percentile curves as a screening tool to predict cardiovascular risk factors and metabolic syndrome risk in Brazilian children. Cad. Saude. Publica..

[45] McCarthy H.D. (2006). Body fat measurements in children as predictors for the metabolic syndrome: Focus on waist circumference. Proc. Nutr. Soc..

[46] Ochoa Sangrador C., Ochoa-Brezmes J. (2018). Waist-to-height ratio as a risk marker for metabolic syndrome in childhood. A meta-analysis. Pediatr. Obes..

[47] Mi J., Munkonda M.N., Li M., Zhang M.X., Zhao X.Y., Fouejeu P.C., Cianflone K. (2010). Adiponectin and leptin metabolic biomarkers in chinese children and adolescents. J. Obes..

[48] Srikanthan K., Feyh A., Visweshwar H., Shapiro J.I., Sodhi K. (2016). Systematic Review of Metabolic Syndrome Biomarkers: A Panel for Early Detection, Management, and Risk Stratification in the West Virginian Population. Int. J. Med. Sci..

[49] Stroescu R.F., Mărginean O., Bizerea T., Gafencu M., Voicu A., Doroș G. (2019). Adiponectin, leptin and high sensitivity C-reactive protein values in obese children—Important markers for metabolic syndrome?. J. Pediatr. Endocrinol. Metab..

[50] Pyrzak B., Ruminska M., Popko K., Demkow U. (2010). Adiponectin as a biomarker of the metabolic syndrome in children and adolescents. Eur. J. Med. Res..

[51] Klünder-Klünder M., Flores-Huerta S., García-Macedo R., Peralta-Romero J., Cruz M. (2013). Adiponectin in eutrophic and obese children as a biomarker to predict metabolic syndrome and each of its components. BMC Public. Health.

[52] Magaña Gomez J.A., Moreno-Mascareño D., Angulo Rojo C.E., de la Peña G.D. (2020). Association of Total and High Molecular Weight Adiponectin with Components of Metabolic Syndrome in Mexican Children. J. Clin. Res. Pediatr. Endocrinol..

[53] Liu Z., Liang S., Que S., Zhou L., Zheng S., Mardinoglu A. (2018). Meta-Analysis of Adiponectin as a Biomarker for the Detection of Metabolic Syndrome. Front. Physiol..

[54] Madeira I., Bordallo M.A., Rodrigues N.C., Carvalho C., Gazolla F., Collett-Solberg P., Medeiros C., Bordallo A.P., Borges M., Monteiro C. (2017). Leptin as a predictor of metabolic syndrome in prepubertal children. Arch. Endocrinol. Metab..

[55] Barbalho S.M., Oshiiwa M., Sato Fontana L.C., Ribeiro Finalli E.F., Paiva Filho M.E., Machado Spada A.P. (2017). Metabolic syndrome and atherogenic indices in school children: A worrying panorama in Brazil. Diabetes Metab. Syndr..

[56] Hromnats’ka N.M. (2014). Types of dislipidemia in children with metabolic syndrome. Wiad. Lek..

[57] Börnhorst C., Russo P., Veidebaum T., Tornaritis M., Molnár D., Lissner L., Marild S., De Henauw S., Moreno L.A., Intemann T. (2019). Metabolic status in children and its transitions during childhood and adolescence-the IDEFICS/I.Family study. Int. J. Epidemiol..

[58] Liang J., Fu J., Jiang Y., Dong G., Wang X., Wu W. (2015). TriGlycerides and high-density lipoprotein cholesterol ratio compared with homeostasis model assessment insulin resistance indexes in screening for metabolic syndrome in the chinese obese children: A cross section study. BMC Pediatr..

[59] Katsa M.E., Ioannidis A., Sachlas A., Dimopoulos I., Chatzipanagiotou S., Rojas Gil A.P. (2019). The roles of triglyceride/high-density lipoprotein cholesterol ratio and uric acid as predisposing factors for metabolic syndrome in healthy children. Ann. Pediatr. Endocrinol. Metab..

[60] Gasevic D., Frohlich J., Mancini G.J., Lear S.A. (2014). Clinical usefulness of lipid ratios to identify men and women with metabolic syndrome: A cross-sectional study. Lipids Health Dis..

[61] Sonmez A., Yilmaz M.I., Saglam M., Unal H.U., Gok M., Cetinkaya H., Karaman M., Haymana C., Eyileten T., Oguz Y. (2015). The role of plasma triglyceride/high-density lipoprotein cholesterol ratio to predict cardiovascular outcomes in chronic kidney disease. Lipids Health Dis..

[62] Vekic J., Zeljkovic A., Cicero A.F.G., Janez A., Stoian A.P., Sonmez A., Rizzo M. (2022). Atherosclerosis Development and Progression: The Role of Atherogenic Small, Dense LDL. Medicina.

[63] Hsu C.N., Hou C.Y., Hsu W.H., Tain Y.L. (2021). Early-Life Origins of Metabolic Syndrome: Mechanisms and Preventive Aspects. Int. J. Mol. Sci..

[64] Wicklow B.A., Becker A., Chateau D., Palmer K., Kozyrskij A., Sellers E.A. (2015). Comparison of anthropometric measurements in children to predict metabolic syndrome in adolescence: Analysis of prospective cohort data. Int. J. Obes..

[65] de Quadros T.M.B., Gordia A.P., Andaki A.C.R., Mendes E.L., Mota J., Silva L.R. (2019). Utility of anthropometric indicators to screen for clustered cardiometabolic risk factors in children and adolescents. J. Pediatr. Endocrinol. Metab..

[66] Higgins V., Adeli K. (2017). Pediatric Metabolic Syndrome: Pathophysiology and Laboratory Assessment. EJIFCC.

[67] Freeman A., Pennings N. (2021). Insuline Resistance.

[68] Contreras D., González-Rocha A., Clark P., Barquera S., Denova-Gutiérrez E. (2023). Diagnostic accuracy of blood biomarkers and non-invasive scores for the diagnosis of NAFLD and NASH: Systematic review and meta-analysis. Ann. Hepatol..

